# Apolipoprotein L1 High-Risk Genotypes are Associated With Lupus Nephritis Incidence

**DOI:** 10.1016/j.ekir.2026.106344

**Published:** 2026-02-04

**Authors:** Samir Patel, Hadi Rabee, Amrita Ramnarine, Dalvir Kular, Evangelos Kougiouris, Mark D. Russell, Mohammad Al-Agil, Maryam Adas, Chris Wincup, Jonathan Dick, Sam Norton, James Galloway, Patrick Gordon, Kate Bramham

**Affiliations:** 1 School of Immunology & Microbial Sciences, King’s College London, London, UK; 2 Medical Subspecialties Institute, Cleveland Clinic London, London, UK; 3 Department of Basic Medical Sciences, University of Jeddah, Jeddah, Saudi Arabia

**Keywords:** African ancestry, apolipoprotein L1, epidemiology, health disparities, lupus nephritis, systemic lupus erythematosus

## Introduction

Development of lupus nephritis (LN) involves a complex interaction of intrinsic and environmental insults, which generalized categories such as race or ethnicity may oversimplify. Ethnic disparities in LN incidence contribute to disproportionate risks of kidney failure and poorer long-term outcomes among global majority groups with systemic lupus erythematosus (SLE).^1^ There is a vital need to elucidate underlying mechanisms to guide equitable and targeted management for those at the greatest risk of developing LN.

Variants in the apolipoprotein L1 (*APOL1*) gene have been linked to a higher risk of nondiabetic chronic kidney disease and are exclusively found in individuals with recent African ancestry.^2^ However, not all individuals with *APOL1* high-risk genotypes (defined by the presence of 2 risk variant alleles, G1 or G2) develop kidney disease,^3^ suggesting that environmental or disease-specific factors act as additional triggers.

Interferons (IFNs) have been implicated as key mediators in *APOL1* kidney diseases.^4^ Type I IFNs are also central to the pathogenesis of SLE, raising the possibility that IFN-driven mechanisms in SLE precipitate LN among genetically susceptible individuals. Previous studies have demonstrated that *APOL1* high-risk genotypes are associated with kidney failure and collapsing glomerulopathy among African American patients with LN.^5^^,^^6^ However, earlier case-control studies reported no significant association with the development of LN.^7^^,^^8^

As such, it remains unclear whether *APOL1* high-risk genotypes contribute to the initial development of LN in patients with SLE, distinct from their role in driving progression to kidney failure. Our aim was to examine this association among individuals of recent African ancestry with SLE using a case-control design.

A detailed description of the methods is provided in the Supplementary Material. Causal pathways were explored using a directed acyclic graph (Supplementary Figure S1).

## Results

Between August 2022 and April 2025, 92 individuals of African ancestry who met SLE classification criteria (S1) and study eligibility criteria were matched into case-control pairs by age and sex. Baseline characteristics are summarized in Supplementary Table S1. High-risk genotypes were more common in participants with LN than without LN (30% vs. 15%, Supplementary Figure S2). One participant with a high-risk genotype (G1/G2) carried an *APOL1* p.N264K variant and was reclassified as having a low-risk genotype for regression analyses.S2 Two participants died following recruitment, both within 3 years of developing kidney failure.

*APOL1* high-risk genotypes were associated with significantly higher odds of LN compared with low-risk genotypes after adjustment for deprivation and smoking status (odds ratio 3.27, 95% confidence interval 1.02–10.53, Table 1). Within the LN cohort, mean time to LN diagnosis varied numerically across groups (1.0 ± 1.6 years for 2 risk alleles vs. 3.1 ± 3.8 years for no risk alleles; Supplementary Table S2), although differences were not statistically significant. Mean time to kidney failure also differed between groups (5.3 ± 4.1 years for 2 risk alleles vs. 11.5 ± 1.3 years for no risk alleles); however, overall numbers were low in each group (2, 6, and 3 individuals progressed to kidney failure in 0, 1, and 2 risk allele groups, respectively). Patterns of LN histology were generally consistent across risk allele categories, except for class V LN, which was seen in 70% of those with 1 risk allele.

**Table 1:** Conditional logistic regression of *APOL1* high-risk genotypes and lupus nephritis

APOL1 genotype	Crude OR (95% CI)	*P-*value	Adjusted OR (95% CI)	*P*-value
Risk genotypes				
Low-risk genotype	Reference		Reference	
High-risk genotype	3.00 (0.97–9.30)	0.06	3.27 (1.02–10.53)	< 0.05
Number of risk alleles				
0	Reference		Reference	
1	1.41 (0.56–3.53)	0.46	1.74 (0.64–4.75)	0.28
2	3.40 (0.94–12.35)	0.06	4.13 (1.08–15.84)	< 0.05

OR, odds ratio; CI, confidence interval.

Cases and controls were matched 1:1 by age and sex. Covariates within the adjusted model included deprivation quintiles (continuous variable) and smoking status (binary variable).

**Figure 1:**
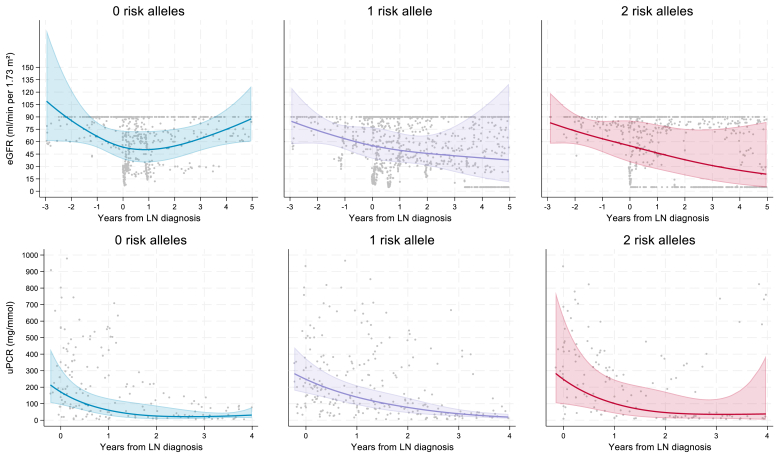
Exploratory geometric mean trajectories of eGFR and uPCR by *APOL1* risk allele groups using mixed-effects models with restricted cubic splines. Measurements are plotted relative to time of renal biopsy (LN diagnosis). N = 40; 0 risk alleles (*n* = 11), 1 risk allele (*n* = 17), and 2 risk alleles (*n* = 12). eGFR, estimated glomerular filtration rates; LN, lupus nephritis; uPCR, urine protein to creatinine ratio.

Longitudinal trends in estimated glomerular filtration rate, urinary protein-creatinine ratio , and serum anti-double-stranded DNA antibody titers were investigated by *APOL1* risk allele categories among 40 participants with LN who had adequate data. Cohort characteristics are provided in Supplementary Table S3. Supplementary Tables S4 and S5 report the number of patients and the number of measurements contributing data to each model. Exploratory analyses suggested differing estimated glomerular filtration rate trajectories across risk allele categories; however, confidence intervals overlapped, and no statistically significant differences were observed between groups (Figure 1). Glomerular filtration slopes are provided in Supplementary Tables S6 and S7. Geometric mean urinary protein-creatinine ratio was elevated at diagnosis across all risk allele groups and declined after LN diagnosis, approaching comparable levels by year 4 (Figure 1, Supplementary Table S6). Anti-double-stranded DNA antibody titers showed no discernible or consistent trends over time across risk allele categories (Supplementary Figure S3).

## Discussion

In this case-control study, we demonstrate an association between *APOL1* high-risk genotypes and LN occurrence in individuals of African ancestry with SLE. Exploratory analyses suggested varying associations with LN and heterogeneity in kidney function trajectories across *APOL1* risk allele categories.

Our findings are in contrast to previous genetic studies that focused on LN occurrence without progression to kidney failure,^7^^,^^8^ although observed differences may reflect variations in methodology. Freedman *et al.*^7^ examined genetic variants in the *APOL1*-neighbouring nonmuscle myosin heavy chain 9 (*MYH9*) gene, initially thought to confer a heightened risk of kidney disease, but did not assess variants in the later-identified *APOL1* gene. Our approach also differed from that of Lin *et al.*,^8^ who included healthy controls without SLE. They reported nominally significant associations between G2 risk alleles and LN in African American patients, which did not meet Bonferroni-adjusted significance thresholds accounting for multiple testing.^8^ By comparing individuals with SLE with and without LN, our study specifically addressed whether *APOL1* high-risk genotypes are associated with LN in people with SLE. This potential association is biologically plausible given the shared IFN-mediated pathophysiology and the overlap among populations at highest risk.

The lack of substantial differences in LN class distribution and focal segmental glomerulosclerosis across risk allele groups in this study suggests an amplifier role for *APOL1* high-risk genotypes, influencing the probability and pace of kidney injury rather than histology. High-risk genotypes may potentiate pre-existing immune mechanisms, increasing susceptibility to LN and accelerating kidney injury once LN occurs. If *APOL1* high-risk variants act downstream of type I IFN signaling, it could lower the threshold for LN to develop, whereas the resulting histological lesion may already be determined by upstream immune drivers. Alternatively, *APOL1* high-risk genotypes may represent an additional vulnerability in those already at risk of LN, reflecting a distinct and independent mechanism of injury.

Although proteinuria is a known risk factor for declining kidney function,^9^ and all risk allele groups had high proteinuria at diagnosis in our study, estimated glomerular filtration rate recovery was observed in the zero-risk allele group. This suggests factors beyond proteinuria, including *APOL1* risk allele status, may influence kidney function trajectories.

This study provides UK-based data on *APOL1* genetic variants in individuals with SLE of recent African ancestry, addressing a paucity of data in Europe. These findings may help explain the disproportionate burden of LN observed in this under-represented population. Integrating *APOL1* genotyping into SLE care could enable earlier identification of those at highest risk for LN and guide precision therapies, such as IFN blockade, to improve outcomes for this vulnerable group.

## Disclosure

KB has received honoraria from Astra Zeneca, Vertex, GSK, Otsuka, and Boehringer Ingelheim and grant funding from Astra Zeneca. JG has received honoraria from Abbvie, Biovitrum, BMS, Celgene, Chugai, Galapagos, Gilead, Janssen, Lilly, Novartis, Pfizer, Roche, Sanofi, Sobi, and UCB and grant funding from Sandoz UK. MDR has received honoraria from AbbVie, Lilly, Galapagos, Menarini, UCB, and Viforpharma; grant funding from Sandoz UK; advisory board fees from Biogen; and support for attending educational meetings from Lilly, Pfizer, Janssen, and UCB. CW has received honoraria from Astra Zeneca, AbbVie, Bristol Myers Squibb, CSL Vifor, Kyverna, Otsuka, UCB; grant funding from Astra Zeneca; and advisory board fees from Biogen. All other authors have no conflict of interest to disclose.

## Supplementary Material

Supplementary File (PDF) **Supplementary Methods. Supplementary References. Figure S1.** Directed acyclic graph illustrating causal pathways from *APOL1* high-risk genotypes (HRG) to lupus nephritis in patients with systemic lupus erythematosus (SLE). **Figure S2.** Distribution of *APOL1* genetic variants by case and control status. **Figure S3.** Geometric mean trajectories of anti-double-stranded DNA (dsDNA) titres by *APOL1* risk allele groups using mixed-effects models with restricted cubic splines. **Table S1.** Baseline characteristics of participants with lupus nephritis (cases) and participants without lupus nephritis (controls) **Table S2.** Characteristics of the lupus nephritis cohort (cases) by number of risk alleles **Table S3.** Characteristics of patients with LN included in mixed-effects model with restricted cubic splines. **Table S4.** Number of participants contributing data towards longitudinal models. **Table S5.** Number of measurements by investigation, year and risk allele groups within longitudinal models. **Table S6.** Predicted geometric mean values and their 95% confidence intervals at 0, 1, and 4 years from mixed-effects model with restricted cubic splines. **Table S7.** eGFR slopes from years one to four after LN diagnosis.

## Footnotes

Supplementary File (PDF)

**Supplementary Methods.**

**Supplementary References.**
**Figure S1.** Directed acyclic graph illustrating causal pathways from APOL1 high-risk genotypes (HRG) to lupus nephritis in patients with systemic lupus erythematosus (SLE) **Figure S2.** Distribution of *APOL1* genetic variants by case and control status. **Figure S3.** Geometric mean trajectories of anti-double-stranded DNA (dsDNA) titres by *APOL1* risk allele groups using mixed-effects models with restricted cubic splines. **Table S1.** Baseline characteristics of participants with lupus nephritis (cases) and participants without lupus nephritis (controls). **Table S2.** Characteristics of the lupus nephritis cohort (cases) by number of risk alleles. **Table S3.** Characteristics of patients with LN included in mixed-effects model with restricted cubic splines. **Table S4.** Number of participants contributing data towards longitudinal models. **Table S5.** Number of measurements by investigation, year, and risk allele groups within longitudinal models. **Table S6.** Predicted geometric mean values and their 95% confidence intervals at 0, 1, and 4 years from mixed-effects model with restricted cubic splines. **Table S7.** eGFR slopes from years 1 to 4 after LN diagnosis
