# Supplementary material for: Apolipoprotein L1 High-Risk Genotypes are Associated With Lupus Nephritis Incidence
Source: Kidney Int Rep. 2026 Feb 4;11(4):106344. doi: 10.1016/j.ekir.2026.106344 (PMC12966666; doi:10.1016/j.ekir.2026.106344)
Supplement: Supplementary File (PDF) — Supplementary Methods. Supplementary References. Figure S1. Directed acyclic graph illustrating causal pathways from APOL1 high-risk genotypes (HRG) to lupus nephritis in patients with systemic lupus erythematosus (SLE). Figure S2. Distribution of APOL1 genetic variants by case and control status. Figure S3. Geometric mean trajectories of anti-double-stranded DNA (dsDNA) titres by APOL1 risk allele groups using mixed-effects models with restricted cubic splines. Table S1. Baseline characteristics of participants with lupus nephritis (cases) and participants without lupus nephritis (controls) Table S2. Characteristics of the lupus nephritis cohort (cases) by number of risk alleles Table S3. Characteristics of patients with LN included in mixed-effects model with restricted cubic splines. Table S4. Number of participants contributing data towards longitudinal models. Table S5. Number of measurements by investigation, year and risk allele groups within longitudinal models. Table S6. Predicted geometric mean values and their 95% confidence intervals at 0, 1, and 4 years from mixed-effects model with restricted cubic splines. Table S7. eGFR slopes from years one to four after LN diagnosis. [file mmc1.pdf]

Supplementary material

Supplementary Methods 2
Supplementary Tables 4
Supplementary Table S1. Baseline characteristics of participants with lupus nephritis (cases) and participants without lupus nephritis (controls). 4
Supplementary Table S2. Characteristics of the lupus nephritis cohort (cases) by number of risk alleles. 5
Supplementary Table S3. Characteristics of patients with LN included in mixed-effects model with restricted cubic splines 6
Supplementary Table S4. Number of participants contributing data towards longitudinal models. . 7
Supplementary Table S5. Number of measurements by investigation, year and risk allele groups within longitudinal models. 8
Supplementary Table S6. Predicted geometric mean values and their 95% confidence intervals at 0, 1, and 4 years from mixed-effects model with restricted cubic splines 9
Supplementary Table S7. eGFR slopes from years one to four after LN diagnosis. 10
Supplementary Figures 11
Supplementary Figure S1. Directed acyclic graph illustrating causal pathways from *APOL1* high-risk genotypes (HRG) to lupus nephritis in patients with systemic lupus erythematosus (SLE). 11
Supplementary Figure S2. Distribution of *APOL1* genetic variants by case and control status. 12
Supplementary Figure S3. Geometric mean trajectories of anti-double-stranded DNA (dsDNA) titres by *APOL1* risk allele groups using mixed-effects models with restricted cubic splines. 13
Supplementary References 14

Supplementary Methods

Study population

We conducted a case-control study nested within a larger observational cohort study (APPLE-CKD: apolipoprotein L1 in people of African ancestry living in the UK: exploration of genetic and environmental factors associated with chronic kidney disease). We recruited participants aged ≥18 years of self-reported African ancestry with SLE fulfilling 2019 EULAR/ACR classification criteria from a regional tertiary referral centre in South London between 2022 and 2025 (Supplementary Reference S1). Cases were defined by the presence of LN on renal biopsy and controls were those with no history of LN or chronic kidney disease (eGFR ≥60 mL/min/1.73m^2 and uPCR <15 mg/mol). Controls were matched 1:1 with cases by age (± three years) and sex.

Data collection

Age, sex, ancestry, Index of Multiple Deprivation (IMD), smoking history, comorbidities (diabetes, hypertension, venous thromboembolic disease, and antiphospholipid syndrome), SLE manifestations, autoantibody status and LN history were recorded. World Health Organization LN class from initial diagnostic biopsies were determined from electronic health records (renal biopsy reports and clinic letters). Investigations were captured from electronic health records using CogStack (www.cogstack.org), including eGFR, uPCR, and serum anti-dsDNA titres. We used both inpatient and outpatient values.

For *APOL1* genetic analysis, DNA was extracted from EDTA blood samples. *APOL1* variant sites were genotyped using a UKAS-accredited assay with TaqMan assay probes (ThermoFisher), G1 (rs60910145, p.I384M, Assay ID C_89555688_30 and rs73885319, p.S342G, Assay ID, C__98253221_10), G2 (rs71785313, p.N388_Y389del, C_102754756_10). High-risk genotypes were defined as those containing two high-risk alleles (G1/G1, G2/G2 or G1/G2); low-risk genotypes were defined as containing zero or one risk allele. We also investigated the presence of an *APOL1* missense variant p.N264K (chr22:36265628 C > A; rs73885316, Assay ID C__98253219_20) which has been shown to protect against the effect of *APOL1* G2 risk alleles, effectively rendering a high-risk genotype (containing a G2 risk allele) to a low-risk genotype (Supplementary Reference S2). Accordingly, participants with *APOL1* high-risk genotypes who carried the p.N264K variant were analysed as low-risk genotypes.

Outcomes

Our primary outcome was the odds of LN occurrence in individuals with *APOL1* high-risk genotypes compared with low-risk genotypes. Secondary outcomes were longitudinal trends in eGFR, uPCR, and serum dsDNA titres according to number of *APOL1* risk alleles.

Statistics

Baseline demographic characteristics were presented for all included individuals. The association between *APOL1* high-risk genotypes (exposure) and LN (outcome) was assessed using conditional logistic regression. Odds ratios (ORs) with 95% confidence intervals were reported. For multivariable analysis, sample size and number of events limited the number of covariates that could be included. Age and sex were accounted for by matching. Based on our directed acyclic graph (Supplementary Figure S1), we selected smoking status (ever-smoker vs non-smoker) and deprivation quintile (modelled as a continuous variable) as the most important confounders for adjustment.

To examine longitudinal trends in eGFR, anti-dsDNA, and uPCR we fitted mixed-effects models with restricted cubic splines (three knots) for time applied to log-transformed values of the outcomes. Values were back transformed for presentation (geometric means). We included interactions between spline terms and number of *APOL1* risk alleles to estimate separate non-linear trajectories by *APOL1* status. Random intercepts accounted for repeated measures within patients and probability weights were applied to ensure equal contribution of each patient regardless of the number of measurements.

Additional covariates were not included in these models to minimise the risk of estimation issues and potential overfitting models given the small sample size. For each analysis, only *APOL1* risk allele groups and years with at least five patients contributing data were included. Supplementary Tables S6 and S7 report the number of patients contributing data and the total number of measurements for each investigation. All available measurements were extracted from hospital electronic health records. Patients with missing data at some time points were not excluded, as the model appropriately accounts for unbalanced data. To improve model accuracy and reflect real-life disease burden, post-kidney failure eGFR measurements were recoded to 5 mL/min/1.73 m^2, while uPCR values after kidney failure onset were excluded due to sampling bias and inaccuracy in renal replacement therapy. EGFR slopes were calculated using model-specified geometric means between years one and four from diagnosis. All statistical analyses were performed using STATA v18 (StataCorp, College Station, TX, USA).

Patient and public involvement

The involvement of patients and participants was integral to shaping the larger APPLE-CKD cohort study. We worked with the Africa Advocacy Foundation to develop the proposal, who also reviewed all patient-facing material.

Ethical approval

The APPLE-CKD study was approved by the Greater Manchester West Research Ethics Committee (REC reference 22/NW/0100 and IRAS reference 292365).

Supplementary Tables

Supplementary Table S1. Baseline characteristics of participants with lupus nephritis (cases) and participants without lupus nephritis (controls). * Values taken prior to lupus nephritis onset for cases. Abbreviations: SLE, systemic lupus erythematosus; SD, standard deviation; VTE, venous thromboembolism; APS, antiphospholipid syndrome; ILD, interstitial lung disease; dsDNA, double stranded DNA; Sm, Smith; RNP, ribonucleoprotein; APL, antiphospholipid.

	**Cases (N=46)**	**Controls (N=46)**
Mean age at recruitment (SD)	40.2 (12.2)	40.6 (12.1)
Mean age at SLE diagnosis (SD)	30.2 (11.8)	29.9 (11.1)
Female sex	44 (96%)	44 (96%)
Ancestry		
African	27 (59%)	17 (37%)
Caribbean	19 (41%)	29 (63%)
Deprivation quintile		
1 (most deprived)	11 (24%)	18 (39%)
2	22 (48%)	16 (35%)
3	8 (17%)	7 (15%)
4	4 (9%)	5 (11%)
5 (least deprived)	1 (2%)	0 (0%)
Smoking		
Non-smoker	39 (85%)	37 (80%)
Ever	7 (15%)	9 (20%)
Comorbidities		
Diabetes*	2 (4%)	2 (4%)
Hypertension*	9 (20%)	12 (26%)
VTE	6 (13%)	5 (11%)
APS	4 (9%)	3 (7%)
SLE manifestations		
Musculoskeletal	35 (76%)	41 (89%)
Skin	31 (67%)	37 (80%)
Mucosa	24 (52%)	26 (57%)
Serositis	23 (50%)	16 (35%)
Myocarditis or ILD	10 (22%)	5 (11%)
Neuropsychiatric	10 (22%)	4 (9%)
Autoantibodies present		
dsDNA	38 (83%)	35 (76%)
Sm	30 (65%)	21 (46%)
RNP	31 (67%)	28 (61%)
Ro	27 (59%)	30 (65%)
≥1 APL antibody	19 (41%)	14 (30%)

Supplementary Table S2. Characteristics of the lupus nephritis cohort (cases) by number of risk alleles. Abbreviations: LN, lupus nephritis; SD, standard deviation; SLE, systemic lupus erythematosus; ESKD, end-stage kidney disease; FSGS, focal segmental glomerulosclerosis; dsDNA, double stranded DNA; Sm, Smith; RNP, ribonucleoprotein; APL, antiphospholipid.

	**Total** (N=46)	**0 risk allele** (N=12)	**1 risk allele** (N=20)	**2 risk alleles** (N=14)
Mean age at LN diagnosis, years (SD)	32.4 (11.9)	33.1 (12.6)	30.6 (14.3)	34.4 (6.6)
Ancestry				
African	27 (59%)	5 (42%)	12 (60%)	10 (71%)
Caribbean	19 (41%)	7 (58%)	8 (40%)	4 (29%)
Mean time to LN from SLE diagnosis, years (SD)	2.2 (3.4)	3.1 (3.8)	2.5 (3.9)	1.0 (1.6)
ESKD	11 (24%)	2 (17%)	6 (30%)	3 (21%)
Mean time to ESKD from SLE diagnosis, years (SD)	8.9 (5.7)	11.5 (1.3)	9.9 (6.7)	5.3 (4.1)
LN histology				
Class I	3 (7%)	0 (0%)	1 (5%)	2 (14%)
Class 2	4 (9%)	1 (8%)	1 (5%)	2 (14%)
Class 3	16 (35%)	5 (42%)	7 (35%)	4 (29%)
Class 4	12 (26%)	4 (33%)	5 (25%)	3 (21%)
Class 5	28 (61%)	6 (50%)	14 (70%)	8 (57%)
FSGS	10 (22%)	2 (17%)	5 (25%)	3 (21%)
Antibodies present				
dsDNA	38 (83%)	9 (75%)	18 (90%)	11 (79%)
Sm	30 (65%)	7 (58%)	14 (70%)	9 (64%)
RNP	31 (67%)	8 (67%)	14 (70%)	9 (64%)
Ro	27 (59%)	6 (50%)	11 (55%)	10 (71%)
≥1 APL antibodies	19 (41%)	5 (42%)	9 (45%)	5 (36%)

Supplementary Table S3. Characteristics of patients with LN included in mixed-effects model with restricted cubic splines. Abbreviations: LN, lupus nephritis; SD, standard deviation; SLE, systemic lupus erythematosus; ESKD, end-stage kidney disease; FSGS, focal segmental glomerulosclerosis; dsDNA, double stranded DNA; Sm, Smith; RNP, ribonucleoprotein; APL, antiphospholipid.

	**Total (N=40)**	**0 risk alleles (N=11)**	**1 risk allele (N=17)**	**2 risk alleles (N=12)**
Mean age at LN diagnosis, years (SD)	34.3 (10.9)	33.7 (13.0)	34.3 (12.2)	35.0 (6.9)
Ancestry				
African	24 (60%)	4 (36%)	11 (65%)	9 (75%)
Caribbean	16 (40%)	7 (64%)	6 (35%)	3 (25%)
Mean time to LN from SLE diagnosis, years (SD)	2.5 (3.5)	3.4 (3.9)	2.9 (4.2)	1.1 (1.7)
ESKD	9 (22%)	2 (18%)	4 (24%)	3 (25%)
Mean time to ESKD from SLE diagnosis, years (SD)	7.4 (5.1)	11.5 (1.3)	7.0 (6.4)	5.3 (4.1)
LN histology				
Class I	3 (8%)	0 (0%)	1 (6%)	2 (17%)
Class 2	3 (8%)	1 (9%)	0 (0%)	2 (17%)
Class 3	14 (35%)	4 (36%)	6 (35%)	4 (33%)
Class 4	11 (28%)	4 (36%)	4 (24%)	3 (25%)
Class 5	25 (62%)	6 (55%)	13 (76%)	6 (50%)
FSGS	10 (25%)	2 (18%)	5 (29%)	3 (25%)
Antibodies present				
dsDNA	32 (80%)	8 (73%)	15 (88%)	9 (75%)
Sm	27 (68%)	7 (64%)	12 (71%)	8 (67%)
RNP	27 (68%)	7 (64%)	12 (71%)	8 (67%)
Ro	24 (60%)	5 (45%)	11 (65%)	8 (67%)
≥1 APLs	17 (42%)	5 (45%)	8 (47%)	4 (33%)

Supplementary Table S4. Number of participants contributing data towards longitudinal models. Data not shown if counts <5.

	Risk allele groups	Year -3	Year -2	Year -1	Year 0	Year 1	Year 2	Year 3	Year 4	Year 5
**Anti-dsDNA**	**0**	-	-	7	9	11	9	7	5	5
	**1**	-	-	8	15	16	14	13	12	11
	**2**	-	-	6	10	11	12	7	7	7
**eGFR**	**0**	5	8	9	10	11	10	7	5	5
	**1**	6	6	9	16	17	14	13	11	11
	**2**	5	5	5	12	11	12	7	7	7
**uPCR**	**0**	-	-	-	10	11	9	7	5	-
	**1**	-	-	-	14	14	13	12	9	-
	**2**	-	-	-	10	10	9	5	5	-

Supplementary Table S5. Number of measurements by investigation, year and risk allele groups within longitudinal models. Counts for data excluded from models were not shown.

	Risk allele groups	Year -3	Year -2	Year -1	Year 0	Year 1	Year 2	Year 3	Year 4	Year 5
**Anti-dsDNA**	**0**	-	-	22	23	30	27	24	19	13
	**1**	-	-	13	41	50	63	46	42	34
	**2**	-	-	12	21	34	35	27	25	25
**eGFR**	**0**	22	66	53	191	155	64	39	40	21
	**1**	46	40	61	185	210	133	92	120	153
	**2**	13	32	30	105	114	108	126	125	143
**uPCR**	**0**	-	-	-	46	48	34	22	18	-
	**1**	-	-	-	50	66	64	45	27	-
	**2**	-	-	-	33	41	24	24	22	-

Supplementary Table S6. Predicted geometric mean values and their 95% confidence intervals at 0, 1, and 4 years from mixed-effects model with restricted cubic splines.

**Investigation**	**Year**	**0 risk allele geometric mean (95% CI)**	**1 risk allele geometric mean (95% CI)**	**2 risk allele geometric mean (95% CI)**
eGFR	0	53.0 (38.5 - 73.0)	54.9 (39.1 - 77.0)	54.8 (35.1 - 85.5)
	1	50.5 (35.1 - 72.7)	49.7 (34.9 - 70.7)	46.4 (26.7 - 80.6)
	4	75.0 (56.6 - 99.4)	40.2 (16.0 - 101.3)	25.5 (8.3 - 78.3)
uPCR	0	163.5 (89.8 - 297.7)	244.4 (162.1 - 368.7)	243.1 (96.8 - 610.8)
	1	61.9 (28.9 - 133.0)	140.7 (93.6 - 211.7)	104.1 (43.4 - 250.3)
	4	30.2 (13.3 - 69.2)	20.1 (10.4 - 38.9)	37.8 (4.0 - 357.3)
dsDNA	0	11.5 (3.1 - 42.2)	25.3 (14.2 - 44.9)	8.9 (3.1 - 25.7)
	1	10.1 (3.0 - 34.5)	22.1 (11.4 - 42.8)	8.3 (3.4 - 20.3)
	4	11.4 (3.2 - 40.0)	17.4 (6.6 - 46.1)	15.0 (4.5 - 50.0)

Supplementary Table S7. eGFR slopes from years one to four after LN diagnosis. Slopes calculated using geometric mean eGFR values derived from mixed-effects models.

	eGFR slope from years one to four after LN diagnosis (mL/min/1.73 m^2/year)
**0 risk alleles (N=11)**	8.2 (-1.3 - 17.7)
**1 risk allele (N=17)**	-3.1 (-18.6 - 12.3)
**2 risk alleles (N=12)**	-7.0 (-21.7 - 7.7)

Supplementary Figures

Supplementary Figure S1. Directed acyclic graph illustrating causal pathways from *APOL1* high-risk genotypes (HRG) to lupus nephritis in patients with systemic lupus erythematosus (SLE).

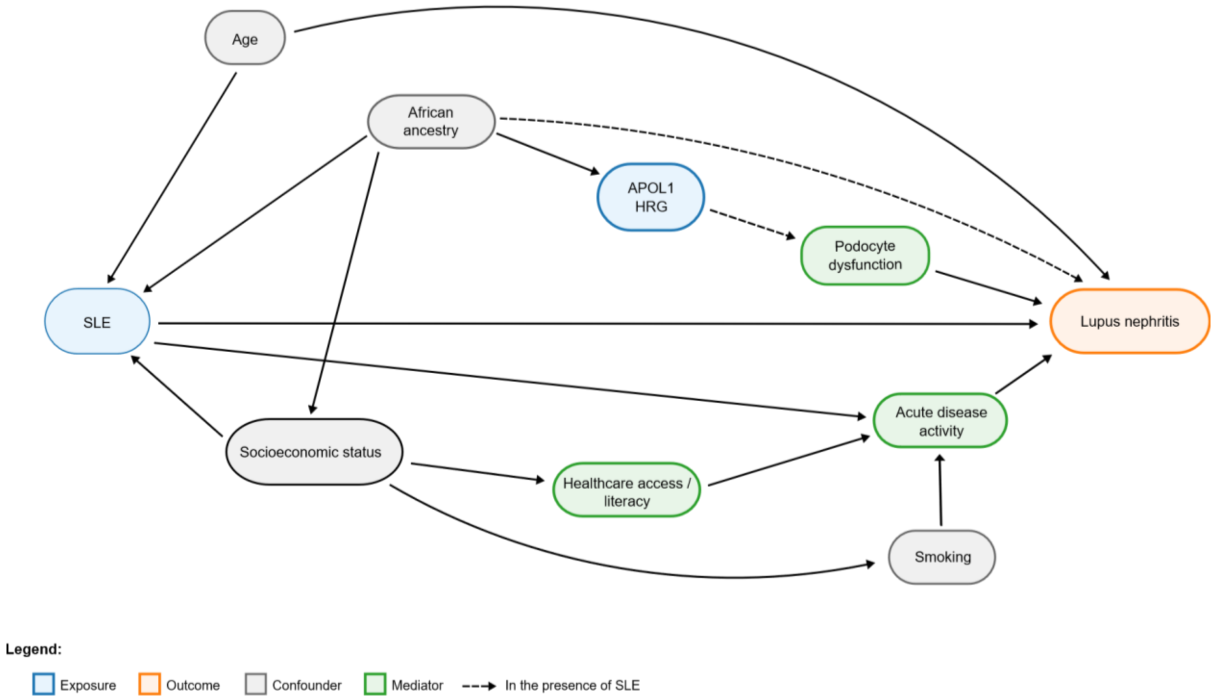

Supplementary Figure S2. Distribution of *APOL1* genetic variants by case and control status. Counts and proportions shown in the table below. *One participant from this group carried an *APOL1* missense variant p.N264K and was reclassified into the low-risk genotype group for regression analyses

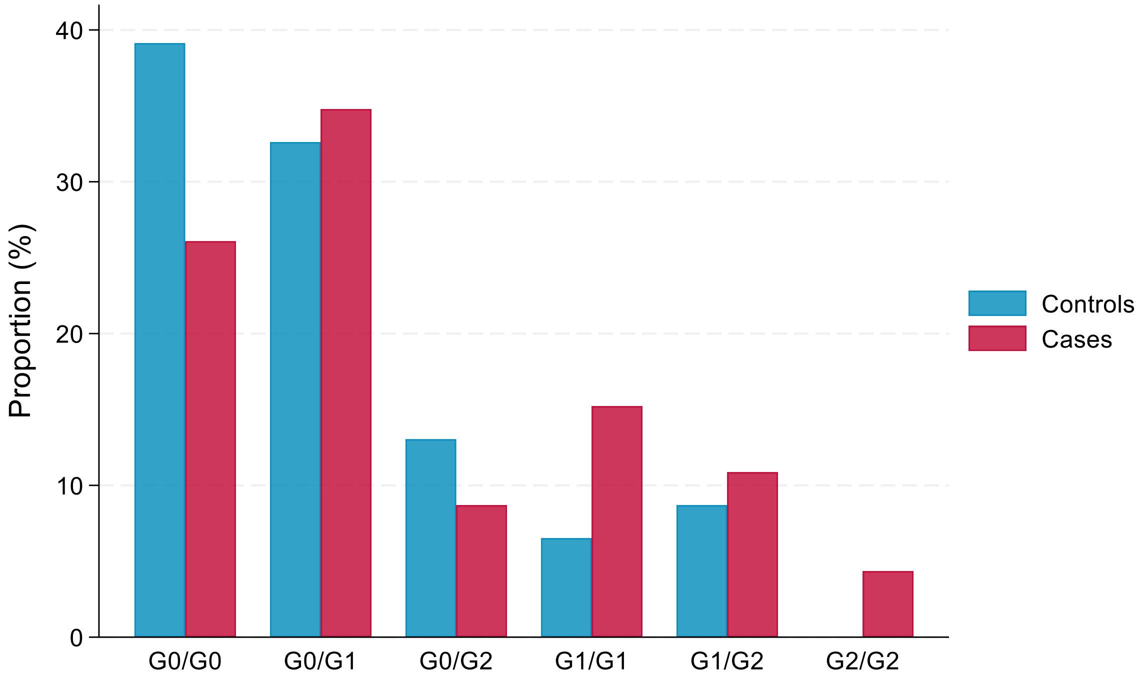

***APOL1* genotypes**	**Controls (N=46)**	**Cases (N=46)**
G0/G0	18 (39%)	12 (26.%)
G0/G1	15 (33%)	16 (35%)
G0/G2	6 (13%)	4 (9%)
G1/G1	3 (7%)	7 (15%)
G1/G2	4 (9%)*	5 (11%)
G2/G2	0 (0%)	2 (4%)

Supplementary Figure S3. Geometric mean trajectories of anti-double-stranded DNA (dsDNA) titres by *APOL1* risk allele groups using mixed-effects models with restricted cubic splines. Measurements are plotted relative to time of renal biopsy (lupus nephritis diagnosis).

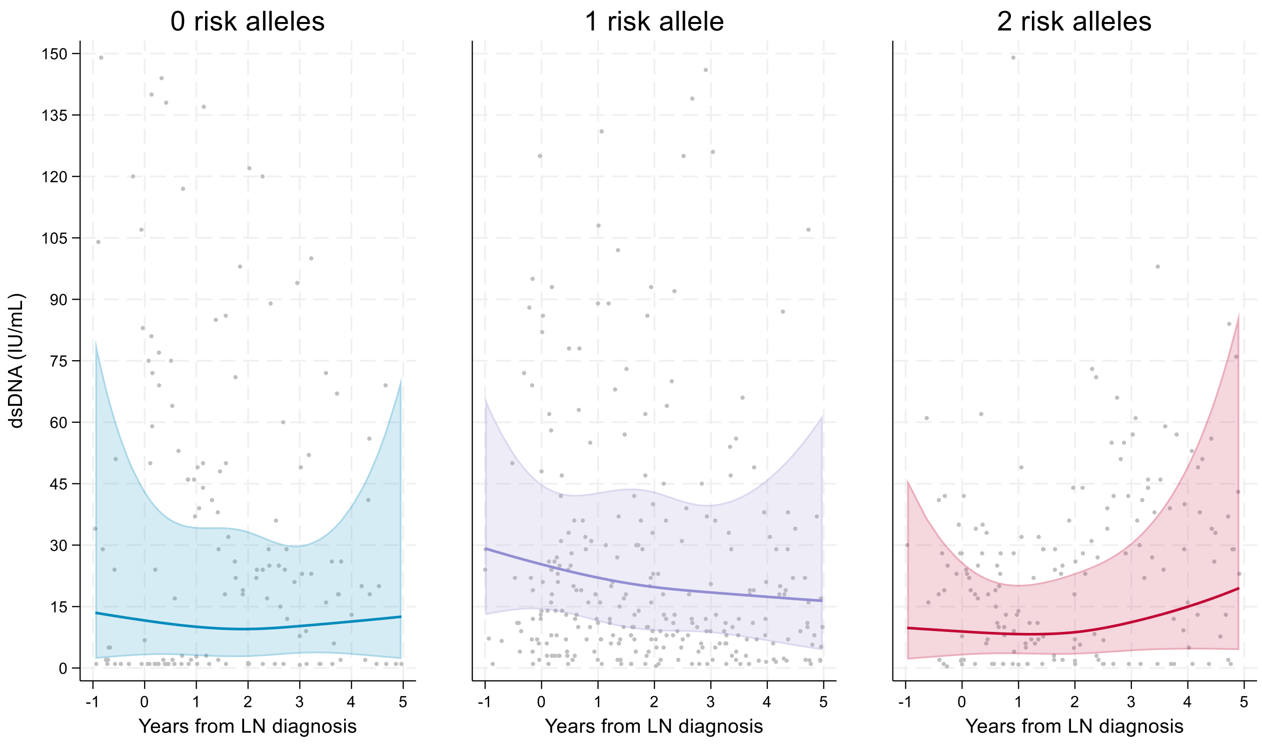

Supplementary References

S1. Aringer M, Costenbader K, Daikh D, Brinks R, Mosca M, Ramsey-Goldman R, et al. 2019 European League Against Rheumatism/American College of Rheumatology classification criteria for systemic lupus erythematosus. Ann Rheum Dis. 2019;78(9):1151-9.

S2. Gupta Y, Friedman DJ, McNulty MT, Khan A, Lane B, Wang C, et al. Strong protective effect of the APOL1 p.N264K variant against G2-associated focal segmental glomerulosclerosis and kidney disease. Nat Commun. 2023;14(1):7836.
